# Dimensional analysis of diffusive association rate equations

**DOI:** 10.1063/5.0238119

**Published:** 2024-11-14

**Authors:** Jixin Chen

**Affiliations:** Department of Chemistry and Biochemistry, Nanoscale and Quantum Phenomena Institute, Ohio University, Athens, Ohio 45701, USA

## Abstract

Diffusive adsorption/association is a fundamental step in almost all chemical reactions in diluted solutions, such as organic synthesis, polymerization, self-assembly, biomolecular interactions, electrode dynamics, catalysis, chromatography, air and water environmental dynamics, and social and market dynamics. However, predicting the rate of such a reaction is challenging using the equations established over 100 years ago. Several orders of magnitude differences between the theoretical predictions and experimental measurements for various systems, from self-assembled monolayers to protein-protein aggregations, make such calculations meaningless in many situations. I believe the major problem is that the time-dependent evolution curve of Fick’s gradient is an ideal assumption in most cases, and its slope is significantly overestimated. This paper digs into Fick’s gradient problem for 3D cases and provides a solution using the single-molecule diffusion probability density function discretely.

## INTRODUCTION TO ASSOCIATION AND COLLISION THEORY

Thermodynamics and kinetics rule our chemistry world, which are often two inseparable aspects of interactions. In kinetics, the two-party reaction is one of the major fundamental unit reactions, e.g., molecules A and B react to produce P = AB,^1–3^

$$\text{A}+\text{B}\overset{k_{f}}{\underset{k_{b}}{\Leftrightarrow }}\text{P},$$ (1)

where *k*_f_ and *k*_b_ are the forward and backward reaction rate constants. At equilibrium,

$$\text{A}+\text{B}\overset{K_{a}}{\underset{K_{d}}{\Leftrightarrow }}\text{P},$$ (2)

*K*_a_ is the binding, affinity, or association equilibrium rate constant, and *K*_d_ is the dissociation constant,

$$K_{a}=\frac{1}{K_{d}}=\frac{k_{f}}{k_{b}}.$$ (3)

**FIG. 1.**
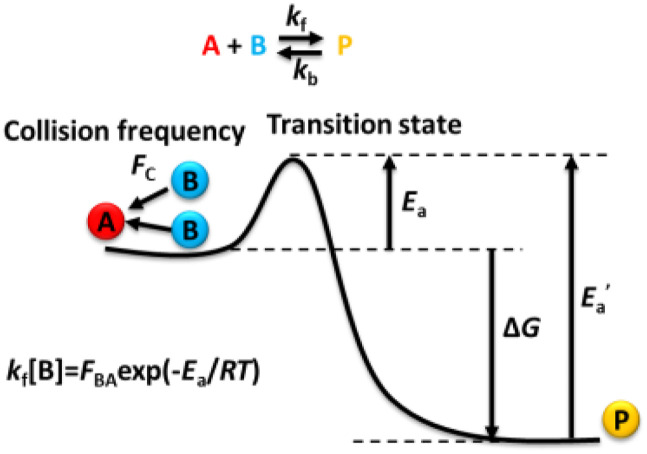
The energy diagram of the association reaction.

In this article, we are calculating the rate constant *k*_f_ for this reaction at the single-molecule level in a diluted solution. Before that, let us go over the basis of collision theory in the textbooks first.^1,2^ Please jump to the next section on diffusion if you are familiar with collision theory. For gas reactions, the forward reaction rate has been predicted by collision theory^4^ and transition state theory (Fig. 1) to be linearly correlated with the concentrations of A and B,^1,2,5,6^

$$r=k_{f}\left[\text{A}\right]\left[\text{B}\right]=\left(k_{f}\left[\text{B}\right]\right)\left[\text{A}\right].$$ (4)

For each A molecule, the pseudo-first-order rate constant has an exponential decay dependent on temperature described by the Arrhenius equation,^1,2,7^

$$k_{f}\left[\text{B}\right]=F_{BA}\exp\left(-\frac{E_{a}}{RT}\right),$$ (5)

where *E*_a_ is the molar activation energy, *R* is the ideal gas constant, and *F*_BA_ is the pre-exponential factor. The value of *F*_BA_ is the key parameter we will discuss in this paper. For an energy barrierless reaction, *E*_a_ = 0, and *F*_BA_ = *k*_f_[B].

The physical meaning of *F*_BA_ is the frequency of collision attempts of the molecules B to this A molecule during 1 s (unit No. collision/s) in a reference volume typically 1 (L). Both concentration and volume linearly proportional affect the collision frequency.

At equilibrium, the association rate constant for this reaction equals the equilibrium rate constant *K*_*a*_ and is related to the thermodynamics with the free energy of the reaction (∆*G*) and temperature (*T*),

$$K_{a}C^{\phi }=\frac{k_{f}C^{\phi }}{k_{b}}=\exp\left(-\frac{∆G}{RT}\right),$$ (6)

where *R* is the ideal gas constant. Typically, we like unit liter for volume in chemistry, but using SI unit m^3^ will make the calculations easier to follow. The standard/reference concentration $C^{\phi }$ = 1 mol/L = 1000 mol/m^3^.

In a diluted solution, the average distance between molecules can be approximated to

$$L\approx \frac{1}{\sqrt[{3}]{C}},$$ (7)

where *C* is the concentration of A, B, or A and B combined, best with unit No. /m^3^ such that *L* is in unit m.

**FIG. 2.**
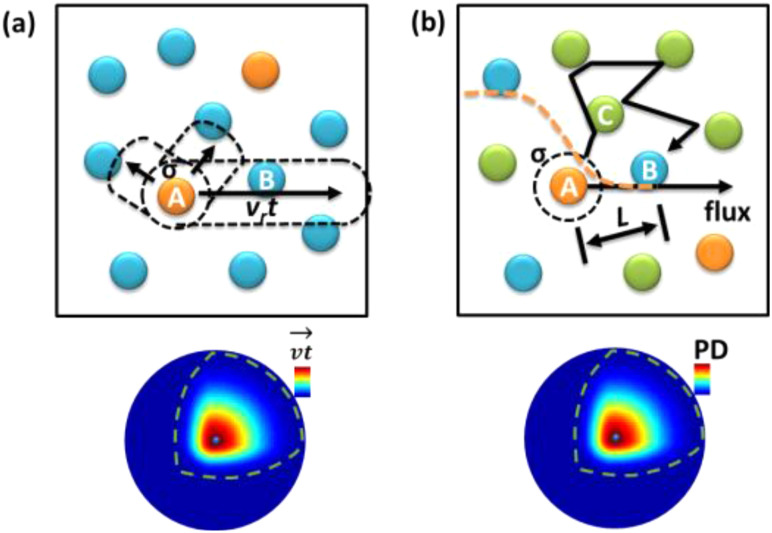
Scheme of (a) collision theory and (b) diffusion theory for association reaction kinetics. All molecules are moving, but relatively speaking, one reactant A is set to be moving, and the other reactant B and solvent C are set to be still in these schemes. The bottom shows the probability density functions of A traveling and diffusing distance at time *t* from the origin of time zero.

In classical collision theory for direct collisions in a mixture of neat gas reactants (no inert gas dilution), the one-to-one collision frequency in the hard-sphere collision model is linearly dependent on the concentration of A and B in the mixture [Eq. (4)]. Assuming A is moving at a relative velocity *v*, B is (relatively) fixed in space, and the collision cross section area is *σ*, the frequency is simply calculated from the average number of B molecules in the path of A molecule traveling in time *t*, creating a virtual volume *σvt* to hold these Bs with concentration *C*_B_ [Fig. 2(a)]. Within this time statistically, this A molecule with all possible speeds and angles spreads out in 3D, yielding a spherical volume with the Maxwell–Boltzmann probability density distribution.^8^ The number of times this A hits a B in duration time *t* is estimated in hard-sphere collision theory,^1,2,4^

$$F_{BA}t=\int_{0}^{\infty }\sigma vtC_{B}4\pi v^{2}{\left(\frac{m}{2\pi k_{B}T}\right)}^{3/2}e^{-\frac{mv^{2}}{2k_{B}T}}dv,$$ (8)

where *σvtC*_*B*_ is the number of B molecules in the hitting zone at a given *v* (scalar) of A and the rest of the equation is the probability of A taking this speed integrated at all angles, *v* is relative scalar velocity, *m* is reduced mass, *k*_B_ is Boltzmann constant, and *T* is temperature.

This model has a linear correlation with time and concentration, i.e., double time doubles the number of collisions, and double concentration doubles frequency. Adding more complicated reaction geometry and activation energy surface on the collision sphere does not affect these correlations that lead to the prediction of rate Eq. (4). We will see if these correlations are held in the diffusive collision models [Fig. 2(b)].

## THEORETICAL MODELS FOR DIFFUSIVE ADSORPTION

In diluted solutions, molecules are doing Brownian motion.^9^ They will not travel straight, and diffusion dominates the molecular transportation [Fig. 2(b)]. Diffusion is usually described by Fick’s 2nd law of diffusion (1855),^10–13^ with the 1D example equation

$$\frac{\partial C\left(x,t\right)}{\partial t}=\frac{\partial }{\partial x}\left[D\left(x,t\right)\frac{\partial C\left(x,t\right)}{\partial x}\right],$$ (9)

where *C* is the solute concentration, *x* is the distance from the origin, *t* is time, and *D* is the diffusion coefficient expressed as a variable in inhomogeneous media in this equation, but usually a constant value is taken in a homogeneous solution. Fick stated in this equation that the concentration profile evolution of a suddenly opened high-concentration solution to pure solvent forms a concentration gradient over time in the space near the interface, resembling the heat flow dynamics published by Joseph Fourier in 1822.^14^ We are interested in 3D problems and will skip real 1D and real 2D diffusion systems in this article.

For Fick’s laws of diffusion in 3D Brownian motion in an ideally still and homogeneous solution, the official solution of the single molecule probability function of a diffusive molecule at time *t* in a small volume at radius *R* from the origin (x, y, *z*) = (0,0,0) is^15–17^

$$\begin{array}{ll} f\left(x,y,z,t\right)dV & ={\left(\frac{1}{4\pi Dt}\right)}^{3/2}e^{-\frac{x^{2}+y^{2}+z^{2}}{4Dt}}dV\\ & =4\pi R^{2}{\left(\frac{1}{4\pi Dt}\right)}^{3/2}e^{-\frac{{\vec{R}}^{2}}{4Dt}}dR, \end{array}$$ (10)

in the radial symmetry spherical coordinate, *dV* = 4 *πR*^2^*dR*. This is a movie of a spherical Gaussian function [Fig. 2(b)] whose peak intensity at origin decreases over time and peak broadens over space. The full width at half maximum of this Gaussian function expands with the square root of *t*, which is different from the collision case where the rate is expended linearly proportional to time [Eq. (8)]. This difference suggests that these two mechanisms (Fig. 2) may predict different rate equations.

Following Fick’s equation, Stokes,^18^ Einstein,^17^ Sutherland,^19^ Smoluchowski,^20^ and many others have used the random walk model to calculate the diffusion coefficient (often a constant), now usually named the Stokes–Einstein or Stokes–Einstein–Sutherland equation^13,16,21,22^

$$D=\frac{k_{B}T}{6\pi \eta r_{0}},$$ (11)

where *k*_B_ is the Boltzmann constant, *T* is temperature, *η* is the viscosity of the solution, and *r*_*0*_ is the radius of the particle. For a molecule approximated to a small ball, *r* can be estimated from the molecular weight $M_{w}=\frac{4}{3}N_{A}\pi r_{0}^{3}\rho$ (kg/m^3^), where *N*_A_ is Avogadro’s number and *ρ* is the density of the neat molecule in the solid or liquid state. All SI units.

The Stokes–Einstein–Sutherland equation has been validated in single-particle and single-molecule measurements.^23^ However, when used in predicting adsorption/association rates, challenges are raised, particularly in calculating the space and time-dependent concentration gradient.^24–26^

Historically, there have been a few equations developed to successfully calculate the adsorption rate of diluted probes to a relatively fixed target molecule at a given probe concentration, for example, the Smoluchowski equation for association in solution^24^ and the Langmuir–Schaefer equation for adsorption to surfaces.^25^ The former deals with spherical Fick’s concentration gradient, and the latter deals with the 1D concentration gradient perpendicular to a surface. Both equations use the classical Fick’s laws of diffusion that can be simulated using a random walk model with Gaussian steps. In this paper, we will stick to the same classical choice that distinguishes us from the abnormal diffusion models.

**FIG. 3.**
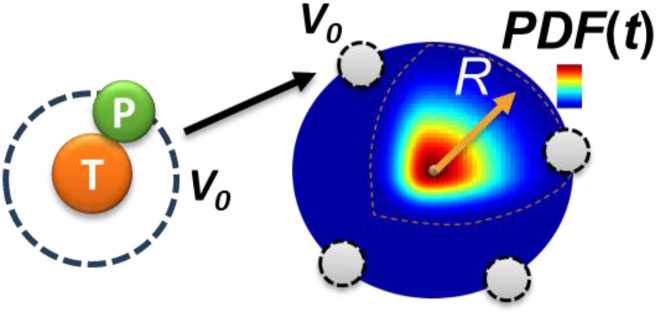
Scheme of collision volume (*V*_*0*_) registered by probes distributed at distance *R* from the origin when a target molecule sits at time zero. These volumes overlap with the diffusing probability density function (PDF) of the target molecule at time *t*.

The most intuitive picture of single-molecule association/adsorption in our mind is to fix the target molecule A still at the origin and let the probes B diffuse and find the target through a random walking search, whose probability functions are evolving as Einstein pictured in Eq. (10). At time *t*, we can fix all the probability functions and calculate how much they overlap with the target molecule. This process registers one possible solution to the original positions of the probes relative to the target. Relatively speaking, it is equivalent to if all probes are fixed over space and the target is diffusing to the probes in which there is only one probability density function. It is easier to calculate the adsorption this way than many functions (Fig. 3). The rate is just the number of B probes over time,

$$r=\frac{1}{t}\int_{0}^{\infty }4\pi R^{2}C_{b}V_{0}{\left(\frac{1}{4\pi Dt}\right)}^{3/2}e^{-\frac{R^{2}}{4Dt}}dR.$$ (12)

*C*_*b*_4*πR*^2^*dR* is the effective number of probes at distance *R* to *R*+*dR* surrounding the target, each having the collision volume *V*_*0*_. Extracting *C*_b_*V*_*0*_ from Eq. (12), the rest is exactly the integral of the normalized Maxwell–Boltzmann distribution that equals 1 [Eq. (10)], meaning a fixed fraction of the sphere contributed to the adsorption. More accurately, we should integrate from *r*_0_ to infinity, but *V*_0_ is very small in diluted solutions, so 0-*r*_0_ can be ignored. The fixed fraction makes sense because the probes are assumed to be uniformly distributed in the solution during this period. Therefore,

$$r=\frac{C_{b}V_{0}}{t}.$$ (13)

Equation (13) is a confusing rate even though the dimensional analysis of unit No. probes/s is correct. The rate is inversely decaying over time, and there is no sensible dependence on the diffusion constant, meaning the speed of diffusion does not matter in this model, which is experimentally wrong. This simple argument ignores the time to establish the concentration gradient that follows Fick’s equation, and the probes are still evenly and discretely distributed in the solution with no concentration gradient. It simply says that eventually there will be a probe hitting the target. Even though the probe as a whole has not arrived, part of its probability function (field) has hit during a time that is set to be continuous. Even if this is true, because different parts of the probe arrive at different speeds, this average speed does not work. We will come back to correct this model later, after we have analyzed a few successful ones.

### A breakthrough

Although irrational, in the above-mentioned picture, we find that the probability of adsorption <1 within any time, no matter how long the time, is due to the imaginary evolution of the concentration gradient when using Fick’s equation. Therefore, it is important to find out the time of relevance. This is because the above diffusion equation assumes that there is only one (pair) molecule and its probability density function is decaying over time at any given space, which is not the case for any systems with multiple molecules. At the moment of one molecule passing the domain of another molecule, time should be restarted as zero because the target molecule cannot distinguish the different probe molecules. Therefore, the imaginary evolution of the concentration gradient should be restarted. This sets the critical probability evolving time *t*_c_ to satisfy the root mean square displacement of diffusion equals the average molecular separation,

$$L=\sqrt{2Dt_{c}}\approx 1/\sqrt[{3}]{C_{b}}.$$ (14)

During this time, the molecules diffuse out from their origins, and the mean displacement of molecules equals the average distance between the molecules in the solution. Therefore, two nearby molecules have a large probability of crossing each other during this time,

$$t_{c}=\frac{1}{2DC_{b}^{2/3}}.$$ (15)

Therefore, the probability density function repeats cycles from fixed 1 at the origin and 0 everywhere else at *t* = 0, to a Gaussian sphere defined by Eq. (10) and then starts a new origin in a stochastic location but on average at their first nearest neighbor distance from the old origin.

In short, the critical time is the first nearest neighbor shuffle time, and the target cannot distinguish the different probes, so as long as the first nearest neighbor is at the same distance on average statistically, everything seems fine to it. It will try a new attempt to establish the imaginary concentration gradient. Most of the time the attempts fail, thus the concentration gradient does not project into the space after the first nearest neighbor layer because these failed attempts do not change anything in the solution real.

If we simulate the movie in the above thought experiment using the average random walk trajectories of each molecule via Monte Carlo simulation, we will find that this discrete picture of recycling in each 0-*t*_c_ period misses the fractal nature of diffusion; in 0-*t*_c_ many smaller steps are self-similar, thus a correction factor is needed to bridge the continuous diffusion with the discrete picture. This factor has been suggested to be 2 from Monte Carlo simulations.^27^

### The 0D equation

**FIG. 4.**
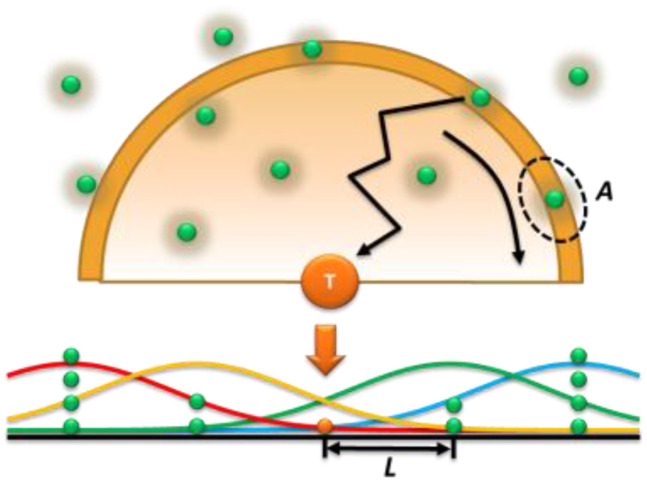
Scheme of stochastic diffusive adsorption of probes to a surface-immobilized target molecule simplified in a 1D random work problem.

With all these settings ready, we can work for solutions mathematically using a discrete model now. One way to solve this problem is to simplify the problem to a 1D random walk problem (Fig. 4).^28^ For a fixed target at origin, we can fold all probes in the solution sphere into a 1D distribution simplified as first neighbor, second neighbor, etc. Note that for an ensemble average, as we discussed for Eq. (12), the probe distribution is continuously quadratic over radius. However, for each given set of targets and probes, the distribution is stochastically discrete, which we draw in Fig. 4 as evenly distributed. This is an acceptable model because only the first few layers matter. We can ignore the layers after the first because the error function is 2 sigma away for the 2nd layer, thus contributions are small. This 1D adsorption rate can be simplified to the effective number of probes (=*a*) in the first neighbor over the critical adsorption time. Multiply the factor of 2 for fractal corrections,

$$\left\langle r\right\rangle =2\frac{a}{t_{c}}=4aDC_{b}^{2/3}.$$ (16)

If there is one effective molecule, the rate of collision is 1/*t*_c_, as the rate is defined as the number of collisions per second. However, this is really attempting frequency when a large percentage of attempts hit empty space, whose fraction is 1-*a*, where *a* will be calculated in a later section. In addition, *t*_c_ is corrected from fractional diffusion to be half the calculated value obtained from Monte Carlo simulations.^27^ This equation also gives the correct unit m^2^ s^−1^(m^−3^)^2/3^ = s^−1^.

### The 2D equations

The geometric information of the adsorption can be used to estimate the effective number of probes. If the adsorption area is *A* (distinguish from *σ* in collision theory) and there are effectively four probes in the first spherical neighbor distance assuming a cubic or tetrahedral packing of the probes in the solution, we can see the effective number is the ratio of the binding area (cross section) of the target-probe pair over the total surface area of the first neighbor sphere,

$$a=\frac{4A}{4\pi L^{2}}.$$ (17)

Substituting Eq. (17) into Eq. (16), we get

$$r=\frac{4}{\pi }A{C_{\text{b}}}^{4/3}D.$$ (18)

**FIG. 5.**
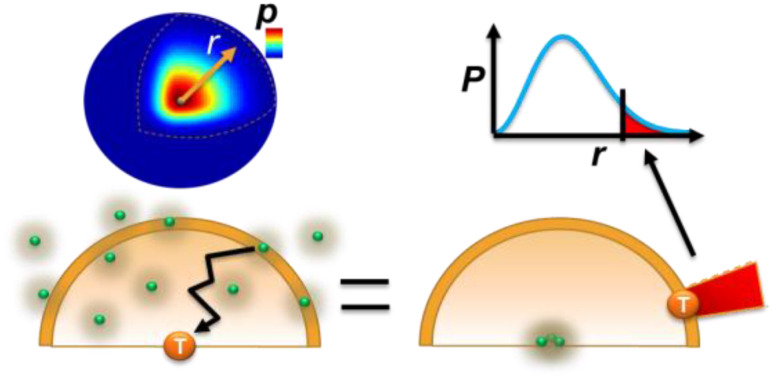
Scheme of integrating the probability density function.

If we like a 3D argument, the effective number of probes can be solved by integrating the probes in the solution with a scheme shown in Fig. 5.^27,29^ This solution has also been solved in the literature by many scientists such as Langmuir and Schaefer,^25^ Ward and Tordai in a continuous model,^26^ or integrate all probes discretely distributed in the solution,^27,29^

$$r=2AC_{\text{b}}\sqrt{\frac{D}{\pi t}}.$$ (19)

Detailed derivation and integration of this Langmuir–Shaefer equation can be found in the literature.^26,27^

Equation (19) is a strange equation with respect to time because the Fick concentration gradient decays over time near the surface and probes are depleted, thus there is a time penalty to the adsorption rate.

The problem with the continuous model is that it assumes that the Fick concentration gradient will project into the next neighbor layers to infinity; therefore, Eq. (19) is suitable for aggregations when the target can accept an infinite number of probes fast enough to create a real Fick’s concentration gradient near it. For example, for diffusive probes to immobilize close-packed arrays of target molecules and pick a small surface area *A* of interest, if the sub-surface concentration gradient is assumed to evolve unlimited over time and space following the Fick’s gradient, the adsorption drops over the square root of time and follows the Langmuir–Schaefer equation [Eq. (19)] simply assuming the surface is an interface that will absorb any probes that hit, and integrate the concentration gradient over space and time from the space- and time-resolved Fick’s equation.^25,26^ The evolution of this real subsurface concentration gradient, however, has been experimentally confirmed to be difficult to predict due to flow and convections in the solution.^25,26^

When we are interested in one-on-one association and ignore the concentration gradient after the first neighbor layer, we can use our critical time *t*_c_ [Eq. (15)] to replace the continuous *t* in Eq. (19), we obtain

$$r=\frac{2\sqrt{2}}{\pi }A{C_{\text{b}}}^{4/3}D.$$ (20)

Which is a factor of square root 2 different from Eq. (17) probably due to the closer packing assumption.

### The 1D equation

There is a dimension between the 0D and 2D approximations of the collision sphere. Using the radius of the collision sphere *r*_0_, Smoluchowski assumed a stable diffusive flux from the probe to the target. Instead of the nearest neighbor boundary condition with a virtual gradient, a real Fick’s gradient will form, yielding^24^

$$r=4\pi r_{0}C_{\text{b}}D,$$ (21)

whose derivation has been detailed in Smoluchowski’s original 1917 seminal papers and has been reviewed in many reviews and textbooks (see Refs. 3 and 30).

This equation using Fick’s gradient and flux assumption is thus suitable for aggregations or crystallizations of probe molecules that form a real concentration gradient near the target. This equation is usually used to define if a reaction has diffusion-controlled kinetics.

### The 3D equation

With the discussion earlier, especially the critical collision time, we can now insert the cutoff time into Eq. (13) and multiply the factor of 2 to correct the fractal diffusion and obtain an equation related to the 3D volume of the collision sphere

$$r=\frac{2C_{b}V_{0}}{t_{c}}=4DC_{b}^{2/3}C_{b}V_{0}=4V_{0}DC_{b}^{5/3},$$ (22)

or equivalently, if we like the folded 1D argument, replace Eq. (17) with

$$a=\frac{C_{b}V_{0}}{\text{unit solution volume}}.$$ (23)

Insert *a* into Eq. (16), and we get the same Eq. (22).

Equation (22) can be rewritten to

$$r=\frac{16r_{0}}{3L}ADC_{b}^{4/3},$$ (24)

which is significantly smaller than Eq. (18) given that *r*_0_ << *L*. This difference comes from the different assumption that in the 3D equation, the probe can cross the target without hitting when they approach each other, thus the probability volume after the target shown in Fig. 5 red zone is not counted into the collision probability. This difference is rather definitive, not mechanistic, because the adsorption rate is a physical value independent of mathematical models. This difference may also come from skipping the fine time details of diffusion and limiting our time resolution to the critical diffusion time of the probes. This blurring introduces uncertainties in the overall adsorption calculations. We believe that adapting different models will affect the estimation of the size of the collision area or volume but will not affect the overall adsorption rate.

### Dimensional analysis of the models

Note that the relative diffusion constant *D* = *D*_A_ + *D*_B_ when both target A and probe B are diffusing.^20,29^

**FIG. 6.**
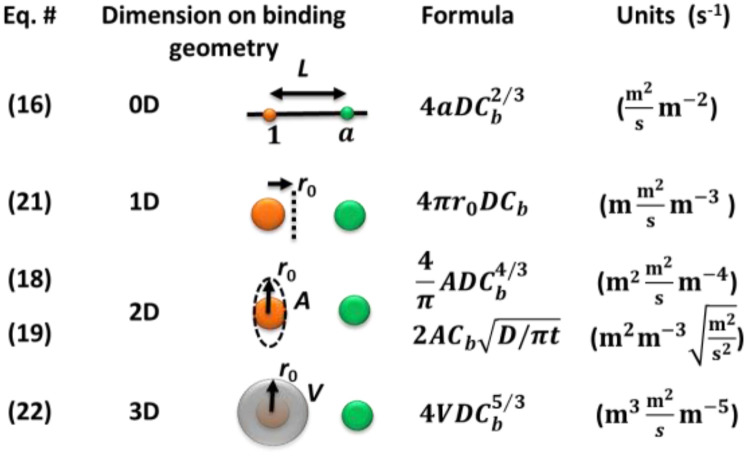
Dimensional analysis of the models.

Equations (16), (18), and (19)–(22) use similar but very different combinations of variables. They all satisfy dimensional analysis to give the correct collision frequency of probes to each target molecule with the unit of rate No. probe s^−1^ (Fig. 6). However, Eqs. (19) and (21) use the boundary condition assuming the concentration gradient continuously evolves at any given time into a real Fick’s gradient, while the other equations reshuffle their virtual Fick gradient at the nearest neighbor diffusion time. They will provide different estimations of the rate over time if they are used for the same reaction system. However, we know that there is only one fact.

Although surprising, the fractional reaction order dependence on the probe concentration is important and interesting because it is significantly different from the pseudo-first-order reaction predicted by a typical kinetic theory from the point of view of the collision theory or Smoluchowski equation.^1–3^ These fractional orders have been observed experimentally in many systems, especially at low concentrations. Comparing Eqs. (8) and (21), we can see collision and diffusion equations share similarities but are different with respect to time. In collision theory, the traveling time is linearly proportional to the separation distance, while in diffusion, it is squarely dependent on the distance. It is unlikely that the two mechanisms share the same reaction order. Therefore, the fractional reaction order, which has been confirmed by the Monte Carlo simulation of fractal diffusion in the literature,^27,31^ requires further experimental confirmation and investigation.

**FIG. 7.**
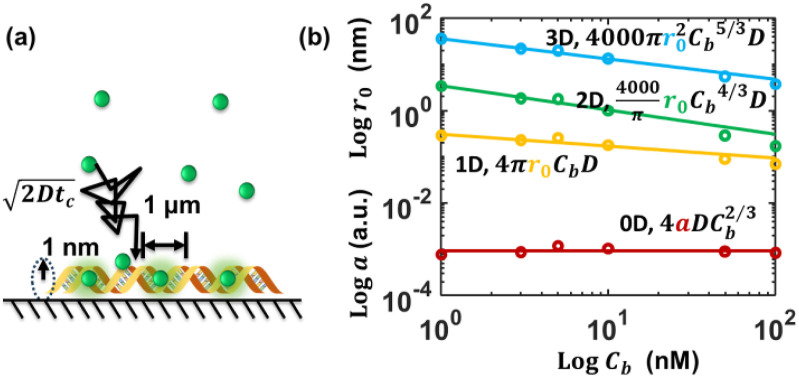
(a) Scheme of the diffusive probe YOYO-1 dye molecules binding to immobilized DNA target molecules. Drawing not to scale. (b) Plot of the fitted parameters in different models as a function of probe concentrations.

We have recently measured the initial diffusive adsorption rate of YOYO-1 dye to fresh 1 *μ*m long flow stretched and immobilized DNA molecules in an aqueous buffer solution.^28^ These data can be analyzed by the different models to obtain the parameters of these models (Tables I and II, Fig. 7). Please see the supplementary material for an example calculation and data fittings.

**TABLE I.** Measured and calculated parameters of the diffusive adsorption system.

Target: Each 1 *µ*m λ-DNA stretched on glass				Probe: YOYO-1 in 10 mM buffer solution				
Model	Radius *r*_0_ (nm)	Area *A* (nm^2^)	Volume *V* (nm^3^)	Model	Mw (g/mol)	Radius (nm)	*D* (m^2^/s)	Debye length (nm)
Cylinder	∼1	∼2000	∼3000	Sphere	1271	∼0.9	2.9 × 10^−10^	∼3

**TABLE II.** Calculated parameters in different adsorption rate equation models.

Input parameters				0D	1D	2D (Eq. (18))		3D (Eq. (22))	
*C*_b_ (nM)	*L* (*μ*m)	*t*_c_ (*μ*s)	*r* (s^−1^)^a^	*a* (×10^−3^)	*r*_0_ (nm)	A (nm^2^)	*r*_0_ (nm)	*V* (×10^4^ nm^3^)	*r*_0_ (nm)
1	1.2	240	0.63	0.77	0.29	3400	3.4	130	36
3	0.8	120	1.49	0.87	0.23	1900	1.9	49	22
5	0.7	83	2.85	1.2	0.26	1800	1.8	40	20
10	0.55	53	4	1.1	0.18	1000	1.0	18	13
50	0.32	18	10	0.90	0.09	290	0.29	3	5.5
100	0.25	11	15	0.85	0.07	170	0.17	1.4	3.8

^a)^ Experimentally measured using a single-molecule fluorescence microscope.^28^

The apparent dependence of the initial binding rate with concentration is ∼2/3 order for this set of data. The 0D model [Eq. (16)] fits the data with a constant correction factor *a* (Fig. 7). This order is apparent, not unit reaction order, because the DNA staining is a combination of multi-step sequential reactions. The rest of the models can factor the different concentration dependence in different steps into the change of the binding radius to make the overall order 2/3. The 1D model significantly underestimates the collision radius, and the 3D model significantly overestimates the volume of the collision spheres. If we reduce the average distance of the first nearest neighbor to ∼*L*/3, *L*/2, or introduce a factor of ∼0.1 correction, the 3D model can obtain a reasonable fitting to the data. The 2D model and associated 0D model correctly estimated the binding geometry for these DNA staining experiments. The reduction of the effective area over the increase of probe concentration is due to the competition of the binding and intercalation. Only intercalated dyes illuminate. Therefore, the higher the concentration, the smaller the ratio of adsorbed dyes that get intercalated and are detected.

## SUMMARY

In summary, the commonly used diffusion-controlled reaction kinetics is misleading because almost all reactions in solutions are diffusion-controlled over time. Even a neat reaction will be controlled by diffusion when the products are significant. In the literature, a series of different equations have been developed to treat different approximations of both the binding geometry and Fick’s concentration gradient for 3D systems over time. We analyze them in this paper using the dimensional analysis method to reveal their underlying assumptions. We focus on 3D diffusion systems and skip real 1D (e.g., kinesin walking problem) and real 2D (e.g., diffusion in lipid bilayers or on a surface) diffusion systems. They can be solved by integrating corresponding diffusion equations or probability density functions, following the same concept as the 3D cases but with different units on concentrations.

In the dimensional analysis, when the adsorption rate of many probes to one target (3D distributed) is set to be a pseudo-first-order reaction respected to the target molecule, the rate of adsorption is in unit No. probes s^−1^. The unit of diffusion constant is m^2^ s^−1^. We can use the number concentration of the probes, the radius, the area, or the volume of the collision sphere to construct the adsorption rate Eqs. (16), (18), (19)–(22), respectively, all satisfying dimensional analysis. For the same 3D distributed many probes in the solution binding to a relatively fixed one target molecule in the solution or immobilized, these equations represent the 0D, 1D, 2D, and 3D geometry of the collision spheres in which the binding sites of the two molecules touch each other. The discrete Eqs. (16), (18), (20), and (22) use the same boundary condition to set a reshuffle time at the nearest neighbor diffusion time of the probes (root mean displacement equals the average neighbor distance). Equations (16), (18), and (20) use the same collision geometry: if the two molecules are close enough, they cannot pass each other and will collide; therefore, they converge to the same equation with respect to the collision area. Equation (22) assumes that the molecules can pass through each other and only the collision volumes matter, thus yielding a smaller prediction than Eqs. (15), (18), and (20) for the same size of collision sphere. The continuous models Eqs. (19) and (21) assume the continuous evolution of the Fick concentration gradient; therefore, they are suitable for aggregations and multi-layer adsorption and represent the lowest estimation of the real binding rate. These two equations have been used to judge if a reaction is diffusion controlled. However, because the actual rate is often orders of magnitude different from these equations predicted, they miscalculate the contribution of diffusion in determining the final rate.

For a given system, the adsorption rate is a fixed experimental value; therefore, each equation has to adjust its parameters to fit the experimental data, yielding a different estimation of the effective size of the collision sphere. If we choose the model in Eq. (18), we will obtain an effective area for a particular pair of molecules, which cannot be used to calculate the effective volume in Eq. (22), and vice versa. Future efforts should be used to unify all models, at least in a narrow time period when the concentration gradient is fixed. All models derived from the snapshot probability density function in this paper assume a factor of two corrections for the fractal diffusion in finer time steps. This factor can be further analyzed in different models. It is obtained from 1D diffusion Monte Carlo simulations that may not be suitable for 2D and 3D models. Updating this factor will affect the standardization of parameters in different adsorption systems.

For the YOYO-1 DNA binding experiment, because the DNA is immobilized on the chemically modified glass surface that is non-sticky to the probes, the surface does alter the probability density function of the probes to the surface, whose effect on the equations requires more careful investigation. Our previous Monte Carlo simulations have suggested a factor of 2 increase compared to half-sphere adsorption from the surface, which is consistent with the mirror effect assumption. However, this doubling means that with or without the substrate does not affect the adsorption rate, which is a confusing result. For more complicated binding systems, rotation and steric effect can be considered.

In summary, the discrete models have significant advantages over the continuous models in calculating single-molecule adsorption rate when the first neighbor concentration is the same as the bulk concentration. When there is a real concentration gradient, the continuous models click in. We can revise the discrete equations to replace the bulk concentration with the estimated probe concentration in the first neighbor sphere to make the discrete model work. Nevertheless, all models must converge with experimental observations because there is only one statistically sound truth for a given reaction.

## SUPPLEMENTARY MATERIAL

The supplementary material encompasses example calculations of and data of Fig. 7 in Excel sheets (xlsx).

## Data Availability

All data are included in the paper and supplementary material.
